# The Addition of Low‐Dose Lidocaine and Triamcinolone Reduces the Adverse Effects of 2‐Deoxycholate Injection Without Affecting the Long‐Term Results

**DOI:** 10.1111/jocd.70072

**Published:** 2025-03-14

**Authors:** Shealinna Ge, Robert A. Weiss, Laura Schilling, Claire M. Noell

**Affiliations:** ^1^ Maryland Laser Skin & Vein Institute Hunt Valley Maryland USA; ^2^ Aesthetic Dermatology Associates Paoli Pennsylvania USA

**Keywords:** cosmetic dermatology, intradermal injection, local adiposity, submental fat

## Abstract

**Introduction:**

The study investigates the efficacy of combining low‐dose triamcinolone acetonide (TAC) and lidocaine with deoxycholic acid (DCA) injections to reduce adverse effects (AEs) without compromising long‐term results.

**Methods:**

A double‐blind, randomized trial was conducted with 15 subjects, divided into a control group (DCA with lidocaine) and an intervention group (DCA with low‐dose TAC and lidocaine). The primary outcomes measured were injection pain, swelling, and submental fat reduction.

**Results:**

Results indicated that the intervention group experienced significantly reduced pain and swelling compared to the control group. Both groups showed similar efficacy in submental fat reduction, with the intervention group demonstrating a greater mean volumetric loss.

**Conclusion:**

The study concludes that the addition of low‐dose TAC and lidocaine to DCA injections is a safe and effective method to enhance patient comfort and treatment outcomes without inducing steroid‐related side effects.

## 1 Introduction

Chin and neck contour has been a subject of frequent concern for patients seeking improvement in facial appearance. A survey by the American Society for Dermatologic Surgery revealed that excess fat in the chin and neck was a significant concern for 67% of the population [1]. Although liposuction and surgical procedures produce the most dramatic results, they may not be suitable for patients seeking to reduce costs and prolonged recovery times. Newer modalities like cryolipolysis, focused ultrasound, radiofrequency, 1060 nm diode laser, cryolipolysis, and injectable deoxycholic acid (DCA) have emerged as additional treatment options. Subcutaneous synthetic DCA (Kybella, Allergan, Madison, NJ) injection was FDA approved for reduction of submental adipose tissue in June 2015 [2]. DCA has been shown to result in long‐term focal destruction of adipocytes without clinically significant increases in serum cholesterol, triglycerides, or free fatty acids in the 24 h following injection [3].

The primary factor preventing DCA widespread adoption has been poor patient experiences. Only 1 in 4 patients return for more than one treatment [4]. More than 70% of patients experience a variable degree of pain, edema, erythema, bruising, induration, and numbness that may persist for several days to weeks following injection [3], which is thought to be a result of immediate irritation due to the acidic nature of DCA, which is an irritating salt. This leads to an immediate local tissue inflammatory response mediated by neutrophil and macrophage infiltration [5]. The long‐term inflammatory response causes fat destruction and removal, but the immediate irritant effect causes unwanted swelling and pain.

Previous groups have explored options to reduce the adverse effect (AE) profile associated with DCA injection. Options like cooling, vibration, topical anesthetics, and NSAIDs [6] have shown success with other cosmetic procedures but may not reduce the prolonged AEs from DCA. In one double‐blind, randomized study coupling triamcinolone acetonide (TAC) with DCA, the addition of 0.5 mL of 40 mg/mL of TAC to 2.0 mL of 2 mg/cm^2^ of DCA reduced the incidence of early edema and tenderness but negatively impacted long‐term efficacy and produced cutaneous changes of steroid atrophy in 40% of the patients treated with TAC [7].

As steroid‐related AEs are often dose‐dependent, further investigations of a similar method, at lower concentrations of TAC, was hypothesized to reduce AEs without compromising treatment efficacy or inducing steroid atrophy. In this study, we examined whether DCA related short‐term AEs can be reduced with the addition of both low‐dose TAC and low‐dose lidocaine without affecting the longer‐term submental fat reduction.

## 2 Methods

### 2.1 Study Design

Patients were evaluated over 11 months at a large dermatology clinic. An independent institutional review board approved the study protocol, and all subjects provided written informed consent according to the Declaration of Helsinki and the International Conference on Harmonization Tripartite Guidelines on Good Clinical Practice.

### 2.2 Subjects

The Clinician‐Reported Submental Fat Rating Scale (CR‐SMFRS), Submental Skin Laxity Grade (SMSLG), and Subject Satisfaction Rate Scale from the REFINE‐1 [8] and REFINE‐2 [9] trials were used for assessments. Patients from 18 to 65 years of age with CR‐SMFRS scores of 2 or 3 were enrolled. Patients were required to have a stable weight (defined as no fluctuations of greater than 15 pounds in a year), diet, and exercise for the preceding 6 months. No subject was taking GLP‐1 agonists. Patients with facial hair were required to remain clean‐shaven for the duration of the study. Subjects were excluded if they had a history of either subcutaneous DCA or other aesthetic treatments (such as focused ultrasound, radiofrequency, botulinum toxin, cryolipolysis, or liposuction) to the chin or neck area within the previous 6 months. Patients who were pregnant or breastfeeding, as well as those with any uncontrolled systemic disease, active infections, wounds, scars, or tattoos within or near the treatment area, were also excluded.

### 2.3 Treatment Protocol

Fifteen subjects were enrolled in this double‐blinded, randomized comparison trial. Randomization was planned in a 2:1 ratio. Ten subjects were randomized to the low‐dose triamcinolone group (LCT group: DCA plus 1 mg/cc TAC and 0.1% lidocaine), and five subjects were randomized to the control group (DCA without TAC but with 0.1% lidocaine). The dosage per 1.0 mL for the control group was 0.8 mL of DCA (10 mg/mL), 0.1 mL of lidocaine 1% without epinephrine, and 0.1 mL of bacteriostatic normal saline. The dosage per 1.0 mL for the LCT group was 0.8 mL of DCA, 0.1 mL of lidocaine 1% without epinephrine, and 0.1 mL of TAC (10 mg/mL). The solutions were mixed in a 1 mL Luer lock syringe with a Luer‐to‐Luer connector. For both groups, the final concentration of DCA was 10 mg/1.2 mL, or 1.6 mg per 0.2 mL injection point.

The treatment area was defined with lines marked superiorly at 1.0 cm inferior to the mandibular margin, laterally by the sternocleidomastoid muscles, and inferiorly by the hyoid bone. Injections were delivered at up to 20 injection points, spaced 1.0 cm apart, with 0.2 mL per injection site, using a 30‐gauge, 0.5‐in. needle. Pre‐treatment chilling with cold packs was used to reduce injection discomfort. No pre‐treatment with injectable lidocaine was permitted to test the efficacy of mixing lidocaine with DCA prior to injection. A maximum total dose of up to 100 mg of DCA was used during this study, which varied based on the number of injection points and the size of the treated area.

Following the baseline questionnaire and evaluations, each subject received a series of three treatments, administered at four‐week intervals. Subjects were asked to score injection pain with each treatment on a 10 point scale 10 min after injections were completed. They were also instructed to return for evaluation 3 and 5 days after each treatment, during which tolerability and AEs were assessed by the subject and investigator using a clinician‐directed, five‐point Side Effects Scale. Following the final injection, additional visits at days 90 and 180 were performed to evaluate safety and efficacy. Subject weight was recorded at baseline and the end of study visits.

**TABLE 1:** Summary of Subject Results. Characteristics and treatment outcomes, baseline versus day 180 of study. Subjects in the control cohort (DCA + 0.1% lidocaine) versus LCT (DCA + TAC 1.0 mg + 0.1% lidocaine) cohort were evaluated at baseline and day 180 of the study. The LCT cohort had a mean volumetric loss of 2.14 cc, as compared to a mean volumetric loss of 0.53 cc for the control group. Volume change was calculated from 3D imaging. Position changes account for those subjects shown as NA. By live assessment, all subjects had reduced submental volume at the conclusion of the study.

Pt	Sex	Age (years)	BMI	Group	Baseline			Day 180				Complications
					CR‐SFRS	PR‐SFRS	SMSLG	CR‐SFRS	SMSLG	SSRS	Volume change (cc)	
1	F	61	30.2	Control	3	2	2	2	2	5	−2.88	None
2	M	62	29.6	TAC	3	3	2	2	2	4	−1.38	None
3	F	51	23.5	Control	3	3	2	1	2	5	NA	None
4	F	58	29.2	Control	2	4	2	1	2	6	+3.29	None
5	F	56	28.3	TAC	3	3	2	1	2	6	+0.43	None
6	F	54	26.6	TAC	3	3	2	1	2	6	−13.68	None
7	F	46	35.8	TAC	2	2	2	2	2	5	−6.61	None
8	F	50	30.2	Control	3	3	2	2	2	4	+1.96	None
9	F	37	30.7	Control	2	3	1	0	1		−4.49	None
10	F	40	24.7	TAC	2	2	1	1	1	6	NA	None
11	F	59	20.5	TAC	2	2	3	0	1	6	+4.75	None
12	F	53	32.6	TAC	2	2	2	2	2	3	−5.11	None
13	F	37	33.6	TAC	3	4	1	3	1	3	+8.66	None
14	F	50	19.6	TAC	2	2	2	1	2	6	−1.75	None
15	F	45	24.3	TAC	2	3	1	2	1	5	−4.61	None

Clinical improvement was evaluated by a blinded investigator using the CR–SMFRS and SMSLG at visit Days 1, 30, 60, 90, and 180. Baseline and final visit 3D images were obtained using a Canfield Vectra H2 system (Canfield Scientific, Parsippany, NJ), and standardized digital photographs were taken at all other visits. Patients graded improvement at the final visit using the SSRS.

## 3 Results

### 3.1 Patient Characteristics and Treatment Outcomes

Fourteen females and one male were enrolled, randomized, and treated (Table 1). The mean (min, max) patient age and BMI were 51.4 (37, 61) years of age and 28.8 (23.5, 30.7) in the control group and 50.2 (37, 62) years of age and 27.6 (19.6, 35.8) in the LCT group. All patients remained within 5 pounds of their starting weight by the end of the study. The mean baseline CR‐SMFRS was 2.6 for the control arm and 2.4 for the LCT arm. Patients rated themselves similarly to CR‐SMFRS ratings at baseline. All patients underwent 3 treatment sessions; one subject in the control group was lost to follow‐up at the final 180‐day visit. The control group received a mean (SD; min, max) dose of 2.67 (0.33; 2, 3) mL per treatment, and the LCT group received a mean (SD; min, max) dose of 2.68 (0.52; 2, 4) mL per treatment.

By the end of the study, the LCT group had a mean volumetric loss of 2.14 cc, and the control group had a mean volumetric loss of 0.53 cc. The number of subjects enrolled did not allow valid statistical analysis, although there was a clear trend toward improved volume loss in the active treatment cohort.

By physician assessment of the LCT cohort, 3 subjects demonstrated a 2‐point improvement, 3 subjects had a 1‐point improvement, and 4 subjects had a 0‐point improvement, with a mean improvement of 0.9 points using CR‐SMFRS. According to patient self‐assessment, the majority were extremely satisfied. According to physician assessment of the control group, two subjects had a 2‐point improvement, and three subjects had a 1‐point improvement. One patient in the control group was extremely satisfied, and three patients were satisfied. Both groups demonstrated minor changes in submental laxity scores, with an overall 0.2‐point improvement in the intervention group, and a 0‐point improvement in the control group.

### 3.2 Injection Pain

Treatment pain was assessed via provided questionnaires. Both groups experienced significantly reduced injection pain for all 3 treatments (*p* < 0.001). There were no differences in volume given at each treatment (*p* = 0.3) and no correlation between volume injected and perceived injection pain (*r* < 0.5) was noted.

### 3.3 Safety

AEs in both the LCT and control cohorts were consistent with those previously reported in randomized clinical trials evaluating DCA. Zero incidences of erosions, ulcerations, steroid‐induced atrophy, marginal mandibular nerve injury, and airway‐obstructing edema secondary to treatments were observed or reported in either group.

The LCT group demonstrated decreased clinician‐assessed tenderness (*p* < 0.05) and patient‐reported pain (*p* < 0.05) 3 and 5 days after the initial treatment, which continued into treatment 2. By treatment 3, there were no significant differences in treatment‐associated tenderness and pain between cohorts.

## 4 Discussion

Loss of mandibular and submental definition associated with increased laxity and fat accumulation superficial and deep to the platysma is a significant cosmetic concern for many patients [10]. Numerous factors, including genetic predispositions, obesity, and other medical comorbidities, may contribute to the propensity for fat accumulation in the chin and neck regions, subtracting from the ideal 90°–105° submental‐cervical angle and exacerbating the undesirable appearance of submental fullness [11, 12]. The percentage of patients seeking cosmetic treatments due to dissatisfaction with their chin and neck area has increased to 73% [13, 14].

Although injectable DCA reduces submental adipose tissue, its use has been significantly limited due to the near‐ubiquitous and often immediate inflammation, swelling, and pain following injection [15]. Not only is DCA an irritant, additional and prolonged tissue inflammation due to local infiltration of neutrophils and macrophages, as well as the persistent presence of mixed lymphocytic infiltrate months post‐treatment, causes prolonged AEs of swelling and pain [5]. Although topical analgesic, tumescent anesthesia, and direct cooling methods have shown some success in mitigating the AEs of DCA [16], these methods are limited by the need for consistent application in daily practice.

Our data shows that the addition of a very low concentration of TAC (1 mg/cc) and a low concentration of lidocaine (0.1%) to DCA prior to injections reduces injection pain and swelling. Both cohorts received injections with a low dose of lidocaine, which contributed to low pain scores for both groups. The LCT group demonstrated greater mean volumetric loss, which may be attributed to the anti‐inflammatory effect of TAC leading to reduced edema. The LCT group also demonstrated larger CR‐SMFRS improvements and superior patient satisfaction compared to controls.

Overall, this study shows that the use of injectable TAC and lidocaine without epinephrine with DCA is safe and well tolerated and does not reduce efficacy or incur any steroid‐related side effects. Addition of low‐dose TAC produced superior results to DCA alone in final treatment outcome measurements. Addition of lidocaine with DCA is highly recommended based on the improvements in pain scores experienced during and after all treatments. Given the near‐universal presence of both TAC and lidocaine in dermatology offices worldwide, their application is readily available for use to enhance tolerability, pain, and results.

Limitations of this study include a small sample size (*n* = 15), variance in group size for the LCT and control groups (*n* = 10 LCT; *n* = 5 control), and inclusion of all Caucasian patients and only one male patient. Evaluation of volumetric change was calculated using 3D images obtained by the Canfield Vectra H2 system, which can be influenced by changes in head and neck positioning as well as movement. Future studies may address limitations by increasing study size in both groups and addressing slight changes in head positioning during image capture.

## Conflicts of Interest

Shealinna Ge, Laura Schilling, and Claire Noell declare no conflicts of interest. Robert Weiss: Allergan/Abbvie, advisory board; Galderma, investigator.

## Data Availability

The data that support the findings of this study are available on request from the corresponding author. The data are not publicly available due to privacy or ethical restrictions.

## References

[1] Surgery ASFD , “American Society for Dermatologic Surgery Consumer Survey on Cosmetic Dermatologic Procedures,” (2015), https://www.asds.net/Portals/0/PDF/consumer‐survey‐2015‐infographic.pdf.

[2] Allergan , “KYBELLA (Deoxycholic Acid) Injection, for Subcutaneous Use,” (2023), https://www.accessdata.fda.gov/drugsatfda_docs/label/2018/206333s001lbl.pdf.

[3] S. H. Dayan , S. Humphrey , D. H. Jones , et al., “Overview of ATX‐101 (Deoxycholic Acid Injection): A Nonsurgical Approach for Reduction of Submental Fat,” Dermatologic Surgery 42, no. Suppl 1 (2016): S263–S270, 10.1097/DSS.0000000000000870. 27787266

[4] S. H. Dayan , J. Schlessinger , K. Beer , et al., “Efficacy and Safety of ATX‐101 by Treatment Session: Pooled Analysis of Data From the Phase 3 REFINE Trials,” Aesthetic Surgery Journal 38, no. 9 (2018): 998–1010, 10.1093/asj/sjy008. 29401213 PMC6094350

[5] R. Thuangtong , J. J. Bentow , K. Knopp , N. A. Mahmood , N. E. David , and M. S. Kolodney , “Tissue‐Selective Effects of Injected Deoxycholate,” Dermatologic Surgery 36, no. 6 (2010): 899–908, 10.1111/j.1524-4725.2010.01566.x. 20482723

[6] S. Fagien , P. McChesney , M. Subramanian , and D. H. Jones , “Prevention and Management of Injection‐Related Adverse Effects in Facial Aesthetics: Considerations for ATX‐101 (Deoxycholic Acid Injection) Treatment,” Dermatologic Surgery 42, no. Suppl 1 (2016): S300–S304, 10.1097/DSS.0000000000000898. 27787270

[7] M. P. Goldman , L. Ishii , R. Zubair , and D. C. Wu , “How We Do It: Subcutaneous Sodium Deoxycholate Injections With or Without Triamcinolone for Reduction of Submental Fat,” Dermatologic Surgery 49, no. 9 (2023): 903–906, 10.1097/DSS.0000000000003856. 37318154

[8] D. H. Jones , J. Carruthers , J. H. Joseph , et al., “REFINE‐1, a Multicenter, Randomized, Double‐Blind, Placebo‐Controlled, Phase 3 Trial With ATX‐101, an Injectable Drug for Submental Fat Reduction,” Dermatologic Surgery 42, no. 1 (2016): 38–49, 10.1097/DSS.0000000000000578. 26673433

[9] S. Humphrey , J. Sykes , J. Kantor , et al., “ATX‐101 for Reduction of Submental Fat: A Phase III Randomized Controlled Trial,” Journal of the American Academy of Dermatology 75, no. 4 (2016): 788–797.e7, 10.1016/j.jaad.2016.04.028. 27430612

[10] B. Rzany , A. Carruthers , J. Carruthers , et al., “Validated Composite Assessment Scales for the Global Face,” Dermatologic Surgery 38 (2012): 294–308, 10.1111/j.1524-4725.2011.02252.x. 22316186

[11] F. B. Naini , M. T. Cobourne , F. McDonald , and D. Wertheim , “Submental‐Cervical Angle: Perceived Attractiveness and Threshold Values of Desire for Surgery,” Journal of Oral and Maxillofacial Surgery 15, no. 4 (2016): 469–477, 10.1007/s12663-015-0872-4. PMC5083688 27833339

[12] O. M. Ramírez , “Advanced Considerations Determining Procedure Selection in Cervicoplasty. Part One: Anatomy and Aesthetics,” Clinics in Plastic Surgery 35, no. 4 (2008): 679–690, 10.1016/j.cps.2008.05.002. 18922321

[13] Surgery ASfD , “American Society for Dermatologic Surgery Consumer Survey on Cosmetic Dermatologic Procedures,” (2018), https://www.asds.net/Portals/0/PDF/consumer‐survey‐2018‐infographic.pdf.

[14] L. Baumann , S. M. Shridharani , S. Humphrey , and C. J. Gallagher , “Personal (Self) Perceptions of Submental Fat Among Adults in the United States,” Dermatologic Surgery 45, no. 1 (2019): 124–130, 10.1097/DSS.0000000000001648. 30234657 PMC6319576

[15] A. M. Rotunda , H. Suzuki , R. L. Moy , and M. S. Kolodney , “Detergent Effects of Sodium Deoxycholate Are a Major Feature of an Injectable Phosphatidylcholine Formulation Used for Localized Fat Dissolution,” Dermatologic Surgery 30, no. 7 (2004): 1001–1008, 10.1111/j.1524-4725.2004.30305.x. 15209790

[16] J. S. Dover , J. M. Kenkel , A. Carruthers , et al., “Management of Patient Experience With ATX‐101 (Deoxycholic Acid Injection) for Reduction of Submental Fat,” Dermatologic Surgery 42, no. Suppl 1 (2016): S288–S299, 10.1097/DSS.0000000000000908. 27787269

